# Sex-related inequalities in current cigarette smoking among adolescents in Africa

**DOI:** 10.1186/s13011-024-00619-5

**Published:** 2024-09-05

**Authors:** Richard Gyan Aboagye, Aliu Mohammed, Precious Adade Duodu, Qorinah Estiningtyas Sakilah Adnani, Abdul-Aziz Seidu, Bright Opoku Ahinkorah

**Affiliations:** 1 School of Population Health, University of New South Wales, Sydney, NSW 2052 Australia; 2 Department of Family and Community Health, Fred N. Binka School of Public Health, University of Health and Allied Sciences, PMB 31, Hohoe, Ghana; 3 Department of Health, Physical Education, and Recreation, University of Cape Coast, Cape Coast, Ghana; 4 Department of Nursing, School of Human and Health Sciences, University of Huddersfield, Queensgate, Huddersfield, England UK; 5 Department of Public Health, Faculty of Medicine, Universitas Padjadjaran, Bandung, West Java Indonesia; 6 College of Public Health, Medical and Veterinary Sciences, James Cook University, Townsville, QLD 4811 Australia; 7 REMS Consultancy Services, Takoradi, Western Region Ghana; 8 Discipline of Psychiatry and Mental Health, School of Clinical Medicine, University of New South Wales, Sydney, Australia

**Keywords:** Adolescents, Africa, Current cigarette smoking, Sex-related inequalities

## Abstract

**Introduction:**

Risky behaviours, including tobacco use, are highly prevalent among adolescents worldwide. Although these behaviours are largely influenced by various sociodemographic factors, including sex, there is a paucity of regionally representative literature on the sex-related inequalities in cigarette smoking among adolescents in Africa. This study examined the sex-based disparities in current cigarette smoking among adolescents aged 13–15 years in Africa.

**Methods:**

The present study employed a secondary analysis of nationally representative data on 45 African countries obtained from the Global Youth Tobacco Survey, accessible through the World Health Organization (WHO) Global Health Observatory. We used the online version of the WHO Health Equity Assessment Toolkit (HEAT) to generate the results.

**Results:**

The prevalence of current cigarette smoking among the adolescents surveyed ranged from 1.6% in Eritrea to 10.4% in Mali among the low-income countries, from 1.3% in Tanzania to 13.1% in Mauritania among the lower-middle-income countries, from 5.2% in Gabon to 15.3% in Mauritius among the upper-middle-income countries, and 14.7% in Seychelles, the only high-income country in the study. The absolute summary measure (D) showed diverse sex-related disparities in the burden of current cigarette smoking among adolescents across the sub-regions. In all countries surveyed, the prevalence of cigarette smoking was higher among male adolescents compared to females, except in Liberia and Mozambique, where female adolescents bore a more significant burden than their male counterparts. Furthermore, male adolescents were more burdened with high cigarette smoking prevalence than females in low-income countries such as Mali, Madagascar, Guinea, Burkina Faso, and The Gambia, where such disparities were most pronounced. Meanwhile, we found less disparity in the burden of cigarette smoking between male and female adolescents in most of the lower and upper-middle-income countries surveyed.

**Conclusion:**

This study sheds light on the sex-based inequalities in the prevalence of current cigarette smoking among adolescents in Africa. In contrast to female adolescents, male adolescents bear a greater burden of current cigarette smoking. The burden of cigarette smoking is most pronounced in low-income countries such as Mali, Madagascar, Guinea, Burkina Faso, and The Gambia. Conversely, in most of the lower and upper-middle-income countries surveyed, the burdens of current cigarette smoking among male and female adolescents were found to be less disparate. Consequently, cigarette smoking prevention programmes and strategies must be implemented in all African nations. There is also the need to intensify interventions aimed at altering the smoking behaviour of male adolescents. Policymakers can develop and implement evidence-based interventions to address the burden of cigarette smoking among the adolescents. Finally, existing policies and programmes addressing adolescents' cigarette smoking should be re-assessed and strengthened to achieve their objectives.

## Introduction

Tobacco use is the leading preventable cause of death worldwide [1]. Globally, tobacco kills more than 8 million people each year, including an estimated 1.3 million non-smokers who are exposed to second-hand smoke [2]. This implies the potential for tobacco epidemic-related health and economic costs to escalate in the coming decades [1, 2]. Tobacco use and exposure have extensive ramifications, including diminished productivity, increased inequality, cancers, and cardiovascular and central nervous system disorders [2–4]. Additionally, the risk of contracting communicable diseases such as tuberculosis (3%), lower respiratory tract infections (4%), and others is increased with tobacco use [5]. There is also an increased risk of addiction to nicotine [6]. However, of great concern is the dangerous effects of tobacco use on the developing brains of children and adolescents [3]. Furthermore, tobacco smokers may have an average life expectancy that is 10 years less than that of people who have never smoked [7, 8].

Unlike most developed countries that have recorded a decreasing prevalence of tobacco use, the prevalence has been increasing in many low- and middle-income countries (LMICs), particularly in sub-Saharan Africa (SSA) [9–11]. Disproportionately, over 80% of the 1.3 billion tobacco users worldwide live in LMICs, which also has the heaviest burden of tobacco-related diseases and deaths [2]. Thus, tobacco use-related deaths in LMICs are predicted to double between 2002 and 2030 [12]. It is noteworthy that low-income countries as well as middle-income countries constitute 10% [13] and 75% [14] of the world’s population of about 8.1 billion [15], respectively. The annual economic burden of tobacco-related illnesses exceeds the total annual health in LMICs [4]. By diverting household spending away from necessities like food and shelter to cigarettes, tobacco usage further contributes to poverty, which is prevalent in LMICs [2].

Tobacco products include cigarettes, roll-your-own tobacco, waterpipe tobacco, heated tobacco, smokeless tobacco, cigars, cigarillos, pipe tobacco, e-cigarettes, bidis, and kreteks. However, the commonest form of tobacco use globally is cigarette smoking [2]. Particularly, the African region is vulnerable to the tobacco industry due to factors such as rising population growth, rising consumer purchasing power, and inadequate implementation of tobacco control laws [9, 16, 17]. For instance, in some African countries, cigarettes are cheaper because they are sold as single sticks irrespective of the provisions in Article 16 of the World Health Organization Framework Convention on Tobacco Control (WHO-FCTC) regarding how it should be sold, thus, increasing the purchasing power of users [18]. Although several African countries have ratified and signed the WHO-FCTC, many are yet to completely implement its strict tobacco control regulations [17]. This lack of implementation of WHO-FCTC’s guidelines has been attributed to governments concentrating on communicable disease threats (issues of competing health priorities), persistent attempts by the tobacco industry to undermine the adoption and enforcement of strict laws (industry influence), corruption, and opposition from the public in some countries because tobacco is seen as their main source of income [19–21]. It is reported that cigarette advertisements are displayed eighty times more in LMICs than in high-income countries [22].

Adolescents (aged 10–19 years) are prone to engaging in risky behaviours such as tobacco use. Furthermore, previous studies have found that initiation of tobacco use occurs predominantly among young people [23–25]. Several factors that are sociodemographic, environmental, and psychosocial have been shown to influence tobacco use among adolescents [26–32]. Most literature attributes the increase in the prevalence of cigarette smoking to aggressive marketing strategies that target young people [9, 33]. However, other contributing factors have been found in previous studies. For example, a study in Saudi Arabia found that peer pressure was a dominant reason for many young people using cigarettes [34]. Furthermore, a recent systematic review on adolescent tobacco use in Nigeria found peer smoking, parental smoking, media advertisements, male gender, increasing age, low parental education, and family economic conditions as significant determinants of tobacco use [35]. Exposure to mass media is positively associated with the odds of tobacco usage among males and females in many African countries [36]. Anxiety [37] and stresses caused by the coronavirus (COVID-19) pandemic [38] have also been cited in recent literature as potential motivators for smoking cigarettes.

Even though the legal age for purchasing cigarettes in most parts of Africa is 18 years, adult smokers are twice as likely to have started smoking at age 13 years or younger than at age 17 years old [39]. Nowadays, adolescents who are in schools around the world consume tobacco at rates of 17% for boys and 15% for girls [40, 41]. Recent research indicates that the rate of increase in cigarette smoking prevalence among females (4.6% to 36.6%) has exceeded that of males (7.8% to 36.5%) [12]. This suggests that there may be a growing trend of female adolescent tobacco use in Africa. Also, the global prevalence of cigarette smoking for adolescents aged 13 to 15 years was found to be 11.3% among boys and 6.1% among girls [40]. These estimates were based on self-reported cigarette smoking at least one day in the previous 30 days. Additionally, prevalence rates among boys and girls were 6.0% (5.5 -6.6) and 2.6% (2.4–2.9) for smoking at least three days, and 4.2% (3.8–4.6) and 1.6% (1.4–1.8) based on smoking at least six days, respectively [40].

Given the significant risk of addiction leading to lifetime smoking, adolescent tobacco use is concerning. Behavioural and biological studies suggest that young people are particularly susceptible to addiction and that most adult smokers regret starting smoking [42–44]. This is a potential motivating standpoint to drive the strategy toward reducing tobacco use among young people. While existing literature has demonstrated an association between sociodemographic characteristics and current cigarette smoking among adolescents in Africa, there is a dearth of regionally representative literature to enable us to critically understand these nuances and how these associations manifest differently based on sex [11, 45]. Therefore, this research investigated the sex-related inequalities in current cigarette smoking among adolescents in Africa. The findings could contribute to the development and implementation of multi-level approaches that prevent young people from starting the use of and becoming addicted to tobacco products. By addressing sex differences and contributing factors in the uptake of cigarette smoking among adolescents, targeted interventions and policies can be developed as part of key strategies to end the tobacco epidemic in Africa.

## Methods

### Data source

Our study was conducted in Africa using data from 45 countries. Data for the study were sourced from the Global Youth Tobacco Survey (GYTS). The data used can be accessed at the World Health Organization’s Global Health Observatory: https://www.who.int/data/gho/data/indicators/indicator-details/GHO/gho-tobacco-control-monitor-survey-reporting-prevalence-of-tobacco-use-or-smoking-among-adolescents. Detailed descriptions of the GYTS, including the sampling methodology and data collection techniques can be found in the literature [46]. In our study, we used the disaggregated dataset incorporated into the WHO’s Health Equity Assessment Toolkit (HEAT) online analytical software to estimate current cigarette smoking [47].

### Measures

The outcome measure of interest was the current cigarette smoking. During the survey, the adolescents were asked to indicate the number of times they had smoked cigarettes in the past 30 days before the data collection using the question “During the past 30 days, how many days did you smoke cigarettes?”. The response options of one or more days were categorized as current users of cigarettes, otherwise, classified as non-users. Sex was the only inequality stratifier available in the WHO HEAT software for examining the disparities in cigarette smoking among adolescents in Africa. The two categories were male and female [47].

### Statistical analysis

We used the WHO HEAT software (online version) for all the analyses [47]. We carried out the analysis in two steps. The first step involved the determination of sex (male and female) estimates of cigarette smoking among adolescents as well as the overall country average. In the second phase, a relative measure of inequality called difference (D) was adopted to show the disparity, and the results were presented in scatter plots. D is a measure of inequality that indicates the difference between two population subgroups on an indicator, which in our study is the difference between females and males on current cigarette smoking. We calculated the values of D by subtracting the rate of cigarette smoking in males from females for each of the countries. In terms of its interpretation, D takes on a value greater than zero, with greater absolute values of D showing a higher inequality in cigarette smoking whereas a zero value showed no evidence of inequality. The scatter plot shows the setting (country) average (on the x-axis) and within-setting inequality as measured by a selected summary measure (on the y-axis). Each country is represented by one coloured circle: benchmark settings are displayed in blue, and the setting of interest is highlighted in orange. Mauritius was used as the benchmark country since it is first in Africa to adopt WHO full-scale tobacco control measures [48]. The results were presented per the United Nations/World Bank classification of countries in Africa. Thus, the countries in Africa were grouped into low-income, lower middle‑income, upper middle‑income and high-income. Seychelles was the only high-income country included in this study.

### Ethical consideration

Ethical clearance was not sought for this study since we analyzed secondary data, which is available in the public domain. However, before the conduct of the GYTS, the WHO and Centers for Disease Control and Prevention (CDC) sought ethical clearance and institutional permission from the health ministries or agencies and the educational ministries in the various countries that participated in the survey. Also, written parental consent and child assent were sought before the adolescents were included in the survey.

## Results

The prevalence of current cigarette smoking among male and female adolescents in low-income, lower-middle-income, and upper-middle/high-income countries in Africa are presented in Tables 1, 2, and 3, respectively.

**Table 1:** Sex-related inequalities in current cigarette smoking in low-income countries

Country	Subgroup	Prevalence (%)	National prevalence (%)
**Burkina Faso**	Female	2.0	6.5
	Male	11.9	
**Burundi**	Female	3.2	4.6
	Male	5.8	
**Central African Republic**	Female	4.3	8.1
	Male	10.4	
**Chad**	Female	4.3	7.5
	Male	8.4	
**Democratic Republic of the Congo**	Female	3.7	8.1
	Male	11.5	
**Eritrea**	Female	0.6	1.6
	Male	2.0	
**Ethiopia**	Female	1.0	2.9
	Male	4.4	
**Gambia**	Female	2.5	6.5
	Male	11.7	
**Guinea**	Female	1.6	7.1
	Male	11.6	
**Guinea-Bissau**	Female	3.0	5.1
	Male	7.2	
**Liberia**	Female	6.4	5.8
	Male	4.6	
**Madagascar**	Female	4.1	8.9
	Male	15.0	
**Malawi**	Female	1.0	3.5
	Male	5.8	
**Mali**	Female	2.5	10.4
	Male	17.4	
**Mozambique**	Female	3.5	2.1
	Male	0.9	
**Niger**	Female	0.6	3.5
	Male	6.8	
**Rwanda**	Female	0.9	1.8
	Male	3.0	
**Sierra Leone**	Female	1.6	3.7
	Male	6.2	
**Togo**	Female	1.2	4.8
	Male	7.4	
**Uganda**	Female	2.4	3.5
	Male	4.7	
**Zambia**	Female	5.7	6.2
	Male	6.2

**Table 2:** Sex-related inequalities in current cigarette smoking in lower-middle-income countries

Country	Subgroup	Prevalence (%)	National prevalence (%)
**Algeria**	Female	0.8	5.7
	Male	12.2	
**Angola**	Female	0.3	2.3
	Male	3.2	
**Benin**	Female	1.3	3.8
	Male	5.1	
**Cameroon**	Female	2.5	5.7
	Male	8.3	
**Comoros**	Female	3.2	6.5
	Male	10.5	
**Congo**	Female	5.0	8.2
	Male	11.3	
**Côte d’Ivoire**	Female	2.1	4.5
	Male	5.8	
**Eswatini**	Female	4.5	6.4
	Male	9.2	
**Ghana**	Female	2.3	2.8
	Male	3.2	
**Kenya**	Female	2.6	4.9
	Male	7.4	
**Lesotho**	Female	7.5	10.1
	Male	11.8	
**Mauritania**	Female	12.0	13.1
	Male	13.4	
**Nigeria**	Female	1.3	3.5
	Male	5.6	
**Sao Tome and Principe**	Female	3.0	4.4
	Male	6.1	
**Senegal**	Female	3.1	4.5
	Male	4.7	
**United Republic of Tanzania**	Female	0.7	1.3
	Male	1.5	
**Zimbabwe**	Female	8.9	11.2
	Male	11.3

**Table 3:** Sex-related inequalities in current cigarette smoking in upper-middle-income and high-income countries

Countries	Subgroup	Prevalence (%)	National prevalence (%)
**Mauritius**	Female	11.5	15.3
	Male	19.6	
**Botswana**	Female	10.9	14.3
	Male	18.1	
**Equatorial Guinea**	Female	3.4	7.0
	Male	9.9	
**Gabon**	Female	4.0	5.2
	Male	6.1	
**Namibia**	Female	6.3	8.1
	Male	10.1	
**South Africa**	Female	11.0	13.0
	Male	15.3	
**Seychelles**	Female	10.3	14.7
	Male	19.6	

### Prevalence of current cigarette smoking among male and female adolescents in low-income

For the low-income countries, we found that the national prevalence of cigarette smoking among adolescents ranged from 1.6% in Eritrea to 10.4% in Mali. Other countries with relatively high prevalence include Madagascar (8.9%), Democratic Republic of the Congo (8.1%), and the Central African Republic (8.1%). The sex-related disparities in the prevalence of current cigarette smoking revealed high prevalence among male adolescents relative to female adolescents in all the low-income countries surveyed, except in Liberia and Mozambique, where the prevalence was higher among female adolescents. Whereas the prevalence of cigarette smoking among male adolescents ranged from 0.9% in Mozambique to 17.4% in Mali, that of female adolescents ranged from 0.6% in Niger and Eritrea to 6.4% in Liberia (Table 1).

### Prevalence of current cigarette smoking among male and female adolescents in lower-middle-income countries

Among the adolescents in lower-middle-income countries, the national prevalence of current cigarette smoking ranged from 1.3% in the United Republic of Tanzania to 13.1% in Mauritania. Other countries with a relatively high prevalence of cigarette smoking among adolescents include Zimbabwe (11.2%), Lesotho (10.1%), and Congo (8.2%). The sex-related disparities in the prevalence of cigarette smoking showed high prevalence among male adolescents relative to female adolescents in all the lower middle-income countries included in the study. While the prevalence of cigarette smoking among male adolescents ranged from 1.5% in the United Republic of Tanzania 13.4% in Mauritania, that of female adolescents ranged from 0.3% in Angola to 12.0% in Mauritania (Table 2).

### Prevalence of current cigarette smoking among male and female adolescents in upper middle-income countries

With regards to the upper middle-income countries, the prevalence of current cigarette smoking among adolescents ranged from 5.2% in Gabon to 15.3% in Mauritius. Other countries with high prevalence include Botswana (14.3%) and South Africa (13.0%). The sex-related disparities in the prevalence of current cigarette smoking revealed high prevalence among male adolescents relative to female adolescents in all the upper middle-income countries surveyed. Whereas the prevalence among male adolescents ranged from 6.1% in Gabon to 19.6% in Mauritius, that of female adolescents ranged from 3.4% in Equatorial Guinea to 11.5% in Mauritius (Table 3).

### Prevalence of current cigarette smoking among male and female adolescents in high income countries

Meanwhile, the only high-income country surveyed, Seychelles, also recorded high prevalence of cigarette smoking among adolescents (14.7%), with higher prevalence observed among males (19.6%) relative to females (10.3%) (Table 3).

**Fig. 1:**
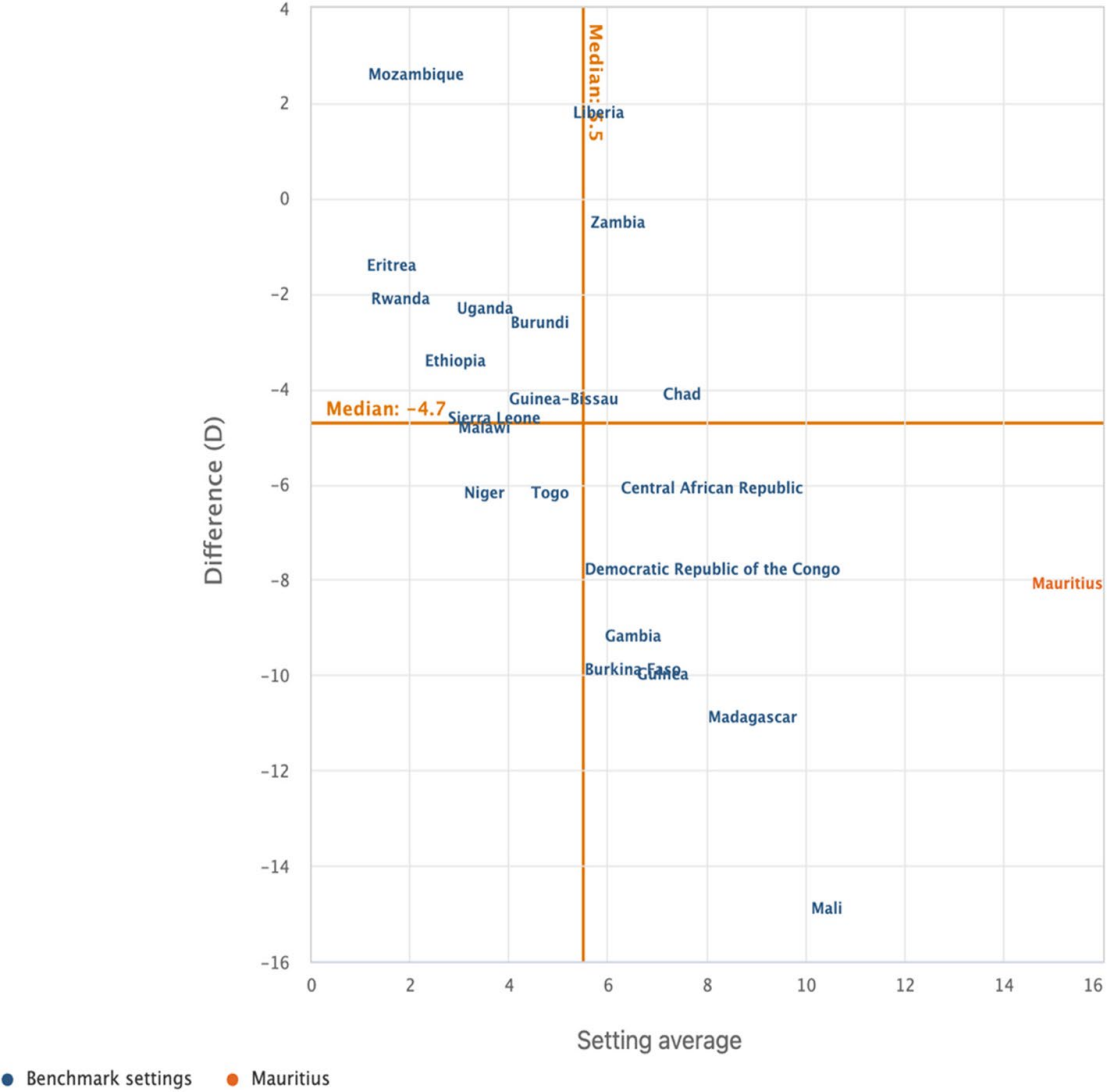
Magnitude of inequality in current cigarette smoking among adolescents by sex in low-income countries

**Fig. 2:**
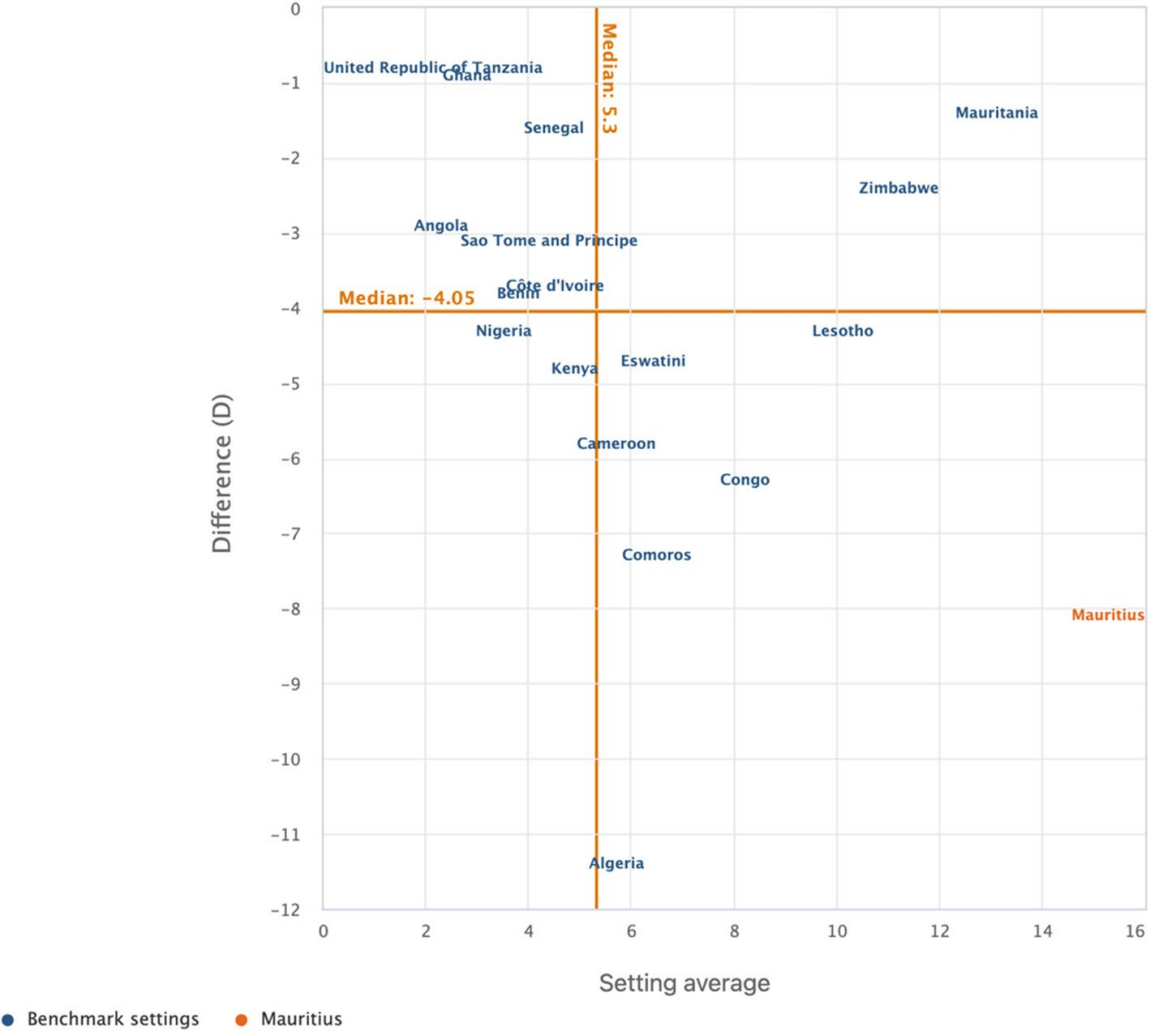
Magnitude of inequality in current cigarette smoking among adolescents by sex in lower-middle-income countries

**Fig. 3:**
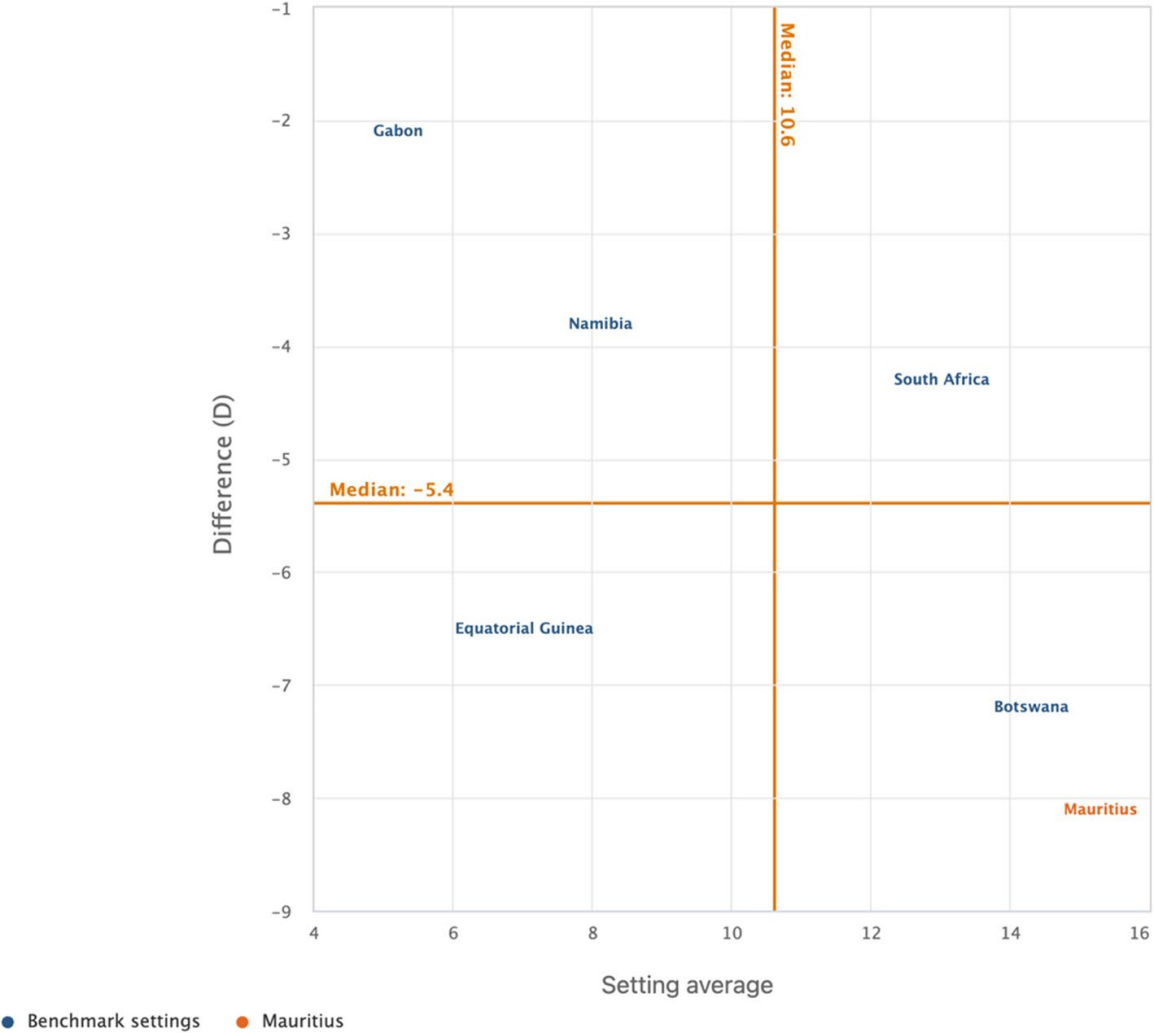
Magnitude of inequality in current cigarette smoking among adolescents by sex in upper-middle-income countries

**Fig. 4:**
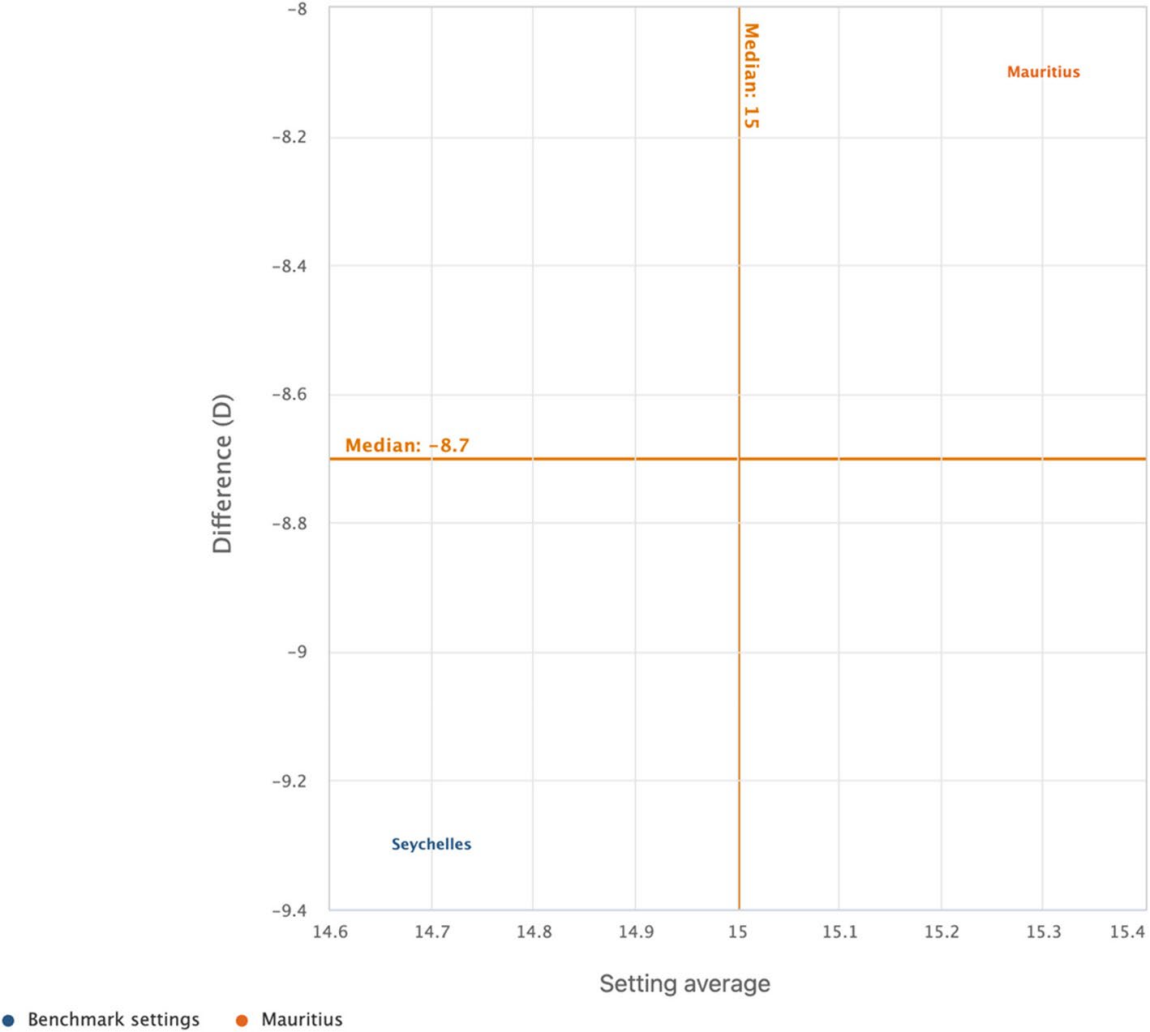
Magnitude of inequality in current cigarette smoking among adolescents by sex in high-income countries

Figures 1, 2, 3, and 4 show the magnitude of inequality in the burden of current cigarette smoking among adolescents based on sex in low-income, lower middle-income, upper middle-income, and high-income countries in Africa, respectively. The absolute summary measure (D) revealed varied sex-related disparities in the burden of current cigarette smoking among adolescents across the sub-regions, with higher burden observed among male adolescents in all the countries surveyed except in Liberia and Mozambique, where the burden was higher among female adolescents. Also, D showed wider disparities in the burden of current cigarette smoking with higher burdens observed among male adolescents, particularly in low-income countries such as Mali, Madagascar, Guinea, Burkina Faso, and The Gambia (Fig. 1). Among adolescents in lower-middle-income countries, only Algeria showed a wider disparity in the burden of current cigarette smoking, with male adolescents having the higher burden (Fig. 2). Similarly, among the adolescents in the upper middle-income countries, only Mauritius showed a wider disparity in the burden of current cigarette smoking, with higher burden observed among male adolescents (Fig. 3). Also, we observed a wider disparity in the burden of cigarette smoking, with male adolescents having higher burden in Seychelles, the only high-income country in Africa (Fig. 4).

## Discussion

In this study, we found that the prevalence of current cigarette smoking among the adolescents surveyed ranged from 1.6% in Eritrea to 10.4% in Mali among the low-income countries, from 1.3% in Tanzania to 13.1% in Mauritania among the lower-middle-income countries, from 5.2% in Gabon to 15.3% in Mauritius among the upper-middle-income countries, and 14.7% in Seychelles, the only high-income country in the study. The sex-related disparities in the burden of current cigarette smoking were largely skewed towards male adolescents in all the countries included in the study, except in Liberia and Mozambique, where female adolescents were the most burdened. Also, we observed wider sex-related disparities in the burden of current cigarette smoking, with higher burden observed among male adolescents, especially in low-income countries like Mali, Madagascar, Guinea, Burkina Faso, and The Gambia. Besides, our findings revealed fewer disparities in the burden of current cigarette smoking between male and female adolescents in all the lower and -upper-middle-income countries, except in Algeria and Mauritius.

In concurrence with the findings from previous studies in Africa [11, 49], our findings revealed a high burden of current cigarette smoking among adolescents which was largely skewed toward male adolescents. Although various sociodemographic, environmental, and psychosocial factors have been associated with increased cigarette smoking among adolescents [27, 30, 32], the high propensity of male adolescents to smoke cigarettes more than their female counterparts has been largely attributed to sociocultural and behavioural factors [33, 50]. Many societies, particularly in LMICs, consider women’s cigarette smoking as an inappropriate and abhorrent behaviour, although smoking among men is largely tolerated [33, 51]. In Nigeria for instance, most communities are tolerant of male smoking while female smoking is largely abhorred [52]. Some Nigerian cultures even demand men to present cigarettes and other tobacco products at certain traditional functions, such as marriages, which tends to increase men’s identity with smoking [52]. This normalisation of the smoking behaviour of men increases the male adolescents’ propensity to smoke [49, 53]. Additionally, compared to female adolescents, male adolescents are generally believed to have a higher tendency to engage in multiple risk behaviours, including cigarette smoking [54]. Perhaps, aside enforcing anti-smoking laws, increasing societal awareness of the dangers associated with cigarette smoking as well as using evidence-based interventions that denormalise cigarette smoking among male adolescents could help in reducing the burden of cigarette smoking among male adolescents in many countries across Africa.

Interestingly, among all the countries studied, only Liberia and Mozambique (low-income countries) showed higher burden of current cigarette smoking among female adolescents relative to their male counterparts. For instance, in Seychelles, the relative measure had a value of -9.3, showing a higher prevalence of cigarette smoking among males compared to females. Similar finding was obtained in South Africa (-4.3) and for the rest of the countries. Contrary to the current findings, a previous study in Liberia reported higher prevalence of cigarette smoking among male adolescents relative to their female counterparts [56]. Also, a recent study in Mozambique reported no significant difference in current cigarette smoking between male and female adolescents [11]. Given the lack of congruence between our findings and the findings from previous studies in both Liberia and Mozambique, there is a need for further comprehensive within-country studies to ascertain the patterns and determinants of current cigarette smoking among female adolescents in Liberia and Mozambique.

We also found that countries with wider sex-related disparities in the burden of current cigarette smoking, with higher burden among male adolescents, were predominantly low-income countries (Mali, Madagascar, Guinea, Burkina Faso, and The Gambia). Several previous studies have reported higher burden of current cigarette smoking among men in low-income countries across all age groups [22, 27, 57]. The increased cigarette smoking among male adolescents in low-income countries has been attributed to the availability and easy accessibility of cigarettes to young people, largely due to regulatory failures and poor implementation of tobacco control programmes [22, 58]. Despite the ratification of the WHO-FCTC by many African countries [11], the tobacco industry is still using aggressive marketing strategies in many African countries, which is often targeted at young people in low-income countries [22]. For example, although Mali ratified the WHO-FCTC in 2006 [55], recent estimates suggest that about 2.7% of adolescent smokers in Mali initiated the behaviour at age 7 years or younger [59], which suggests easy access to cigarette among young people. Therefore, it is important for stakeholders and authorities involved in tobacco law enforcement to intensify monitoring to limit accessibility to tobacco products in Africa, especially in low-income countries.

Furthermore, we observed less sex-related disparities in the burden of current cigarette smoking among adolescents in all the lower and upper-middle-income countries, except for Algeria and Mauritius. This finding suggests that the proportion of current female adolescent cigarette smokers in most of the lower and upper-middle-income countries in Africa may be getting closer to that of their male counterparts. Previous studies have reported that the ratio of female to male cigarette smokers has declined significantly over the past few decades in most high-income countries [60–62], suggesting an influence of socioeconomic development in reducing sex-related disparities in cigarette smoking. Additionally, Amos et al. [60] suggested that there is a growing epidemic of cigarette smoking among female adolescents in LMICs, which might have contributed to the reduced sex-related differences in the burden of current smoking among adolescents in the lower and upper-middle-income countries as observed in the present study. The increasing spate of socioeconomic development and globalisation in Africa, especially in the lower and upper-middle-income countries, has been associated with reducing sex-related gaps in the burden of current cigarette smoking among adolescents [11]. Choi et al. [63] suggested that globalisation is often associated with acculturation, which could influence behavioural changes and may predispose more female adolescents to cigarette smoking. Thus, our findings affirm the raging concern of the potential increase in cigarette smoking among female adolescents in Africa [60]. Therefore, researchers and policymakers across Africa need to intensify research and monitoring of cigarette smoking behaviours of female adolescents in Africa, particularly in the lower and upper-middle-income countries, to design and implement evidence-based interventions to address the problem.

### Practical implications

Although cigarette smoking has generally declined in high-income countries, the behaviour continues to increase in many LMICs, particularly among adolescents in Africa [58]. Given that most cigarette smokers often start smoking between ages 12 and 25 years [64], interventions targeted at preventing cigarette smoking among adolescents could essentially reduce the prevalence of cigarette smoking and its associated burden of diseases. While our findings revealed a high burden of current cigarette smoking among male adolescents relative to their female counterparts in most of the countries studied, we observed a higher burden of male adolescent smoking in predominantly low-income countries including Mali, Madagascar, Guinea, Burkina Faso, and The Gambia. Therefore, whereas cigarette smoking prevention programmes and strategies must be enforced in all African countries, intensifying interventions that focus on changing male adolescents’ smoking behaviour could immensely benefit low-income countries like Mali, Madagascar, Guinea, Burkina Faso, and The Gambia. Other countries that could benefit significantly from male adolescents’ targeted interventions are Algeria, Mauritius, and Seychelles. Meanwhile, the higher burden of current cigarette smoking observed among females relative to male adolescents in Liberia and Mozambique calls for urgent within-country studies to ascertain the patterns and determinants of current cigarette smoking among adolescents in these countries. Also, the less disparity observed in the burden of current cigarette smoking between male and female adolescents in almost all the lower and upper middle/high-income countries seems to suggest an increase in the proportion of female adolescent cigarette smokers, which could be bridging the gap between them and their male counterparts. Hence, there is an urgent need for researchers and policymakers to pay particular attention to cigarette smoking patterns of female adolescents in Africa, especially in the lower and upper-middle/high-income countries, to design and implement evidence-based interventions to address the problem.

### Strengths and limitations

To examine the sex-related disparities in current cigarette smoking among adolescents, we utilized nationally representative data collected from 45 countries across Africa. This approach yielded more dependable information regarding the prevalence of cigarette smoking among male and female adolescents in Africa. Therefore, our results may serve as foundational information for future research comparisons and trend monitoring regarding the present-day smoking habits of male and female adolescents in Africa. Notwithstanding these strengths, our results must be interpreted with specific constraints in mind. Firstly, while it is acknowledged that a variety of contextual elements can impact adolescent cigarette consumption, our analysis was limited to sex alone. The impact of additional contextual factors was not evaluated due to their absence from the datasets utilized in this research. Furthermore, because our analyses were limited to data collected for the most recent survey from multiple countries, we could not conduct a trend analysis. Thus, there is a need for future studies to ascertain the trend of cigarette smoking among male and female adolescents using data from multiple survey years to ascertain the temporal progression of cigarette smoking among adolescents in Africa. Finally, the present smoking habits of the participants were disclosed by themselves, rendering them susceptible to recall and social desirability biases.

## Conclusion

The present study highlights the sex-related disparities in current cigarette smoking among adolescents in Africa. Our findings revealed that male adolescents have a higher burden of current cigarette smoking than their female counterparts, with an increased burden among male adolescents observed mainly in low-income countries like Mali, Madagascar, Guinea, Burkina Faso, and The Gambia. Meanwhile, most of the lower and upper-middle-income countries surveyed showed less disparity in the burden of current cigarette smoking between male and female adolescents. Perhaps the proportion of female adolescent cigarette smokers in Africa could be increasing thus, bridging the gap between them and their male counterparts, particularly in the lower and upper-middle/high-income countries. Therefore, considering the negative repercussions of tobacco use on women’s health, there is the need for urgent investigations to ascertain the cigarette smoking patterns of female adolescents and its associated factors in Africa to implement evidence-based interventions to address the phenomenon. While cigarette smoking prevention programmes and strategies need to be enforced in all countries across Africa, intensifying interventions that focus on changing male adolescents’ smoking behaviour could immensely benefit low-income countries like Mali, Madagascar, Guinea, Burkina Faso, and The Gambia.

## Data Availability

No datasets were generated or analysed during the current study.

## Abbreviations

CDC: Centers for Disease Prevention and Control
COVID-19: Coronavirus Disease 2019
D: Difference
GHO: Global Health Observatory
GYTS: Global Youth Tobacco Survey
HEAT: Health Equity Assessment Toolkit
LMICs: Low-and Middle-Income Countries
SSA: Sub-Saharan Africa
WHO-FCTC: World Health Organization Framework Convention on Tobacco Control
